# Clinician’s and women's perceptions of individual barriers to vaginal birth after cesarean in Iran: A qualitative inquiry

**DOI:** 10.22088/cjim.11.3.259

**Published:** 2020-05

**Authors:** Mahboobeh Firoozi, Fatemeh Tara, Mohammad Reza Ahanchian, Robab Latifnejad Roudsari

**Affiliations:** 1 Nursing and Midwifery Care Research Center, Mashhad University of Medical Sciences, Mashhad, Iran; 2 Ferdowsi University of Mashhad, Mashhad, Iran; 3 Department of Midwifery, School of Nursing and Midwifery, Mashhad University of Medical Sciences, Mashhad, Iran

## Abstract

**Background:**

High rate of repeat cesarean section and its complications are the results of cesarean tsunami in the last two decades in Iran. Vaginal birth after cesarean (VBAC) is an important alternative for repeat cesarean. However, the rate of VBAC in Iran is very low subject to some organizational and individual barriers is very low. This study explored the clinician’s and women's perceptions of individual barriers to achieve VBAC.

**Methods:**

In this conventional content analysis, 28 semi-structured interviews and one focus group discussion was conducted with health care providers including gynecologists, midwives and family physicians as well as prior cesarean section mothers attended one of the women's hospitals in Mashhad, Iran in 2017. Participants were selected through purposive sampling considering the strategy of maximum variation. Data were analyzed according to Graneheim and Lundman (2004) method using MAXQDA.10 software.

**Results:**

The theme of “obstacles to acceptance and committed actions” emerged from two categories of “psychological barriers” and “operational barriers". Psychological barriers included 'sense of danger”, “financial displeasure" and "negative attitude"; whereas, operational barriers consisted of 'barriers to decision making' and 'indolence'.

**Conclusion:**

Improving women's attitude via maternity care promotion, creating supportive environment, informing mothers about choice of birth mode and empowering them in shared decision making could influence women's VBAC request. Also organizing VBAC care team and creating motivations in medical team and hospital directors through reporting of research project outcomes on safety and benefits of VBAC could affect the VBAC rate.

The World Health Organization (WHO) recommends that the rate of cesarean section should not exceed 15% (1), however, the average rate of cesarean section was 50.77% in Iran in 2017. (2). Complications of cesarean section, not only threatened survival of mother and infant, but also raise the expenses of the family by two to three times more. Therefore, in May 2014, the Iran’s Ministry of Health and Medical Education announced the health reform plan promoting natural childbirth package. The first year after implementing this package, a significant reduction (up to 10%) was seen in cesarean section rate in many cities, but then its rate remained almost plateau, because one of the factors influencing the total rate of cesarean section is the rate of repeat cesarean. Institutions and international associations are trying to modify cesarean birth rates by the replacement of VBAC as a safe option (3). National Institute of Health (NIH) (2010) recommended medical community to remove VBAC barriers for women, in order to reduce the total cesarean rate safely (4).

The possibility of cesarean for women who has already given birth vaginally is 10%, while this possibility either as emergency or elective cesarean in the presence of once prior cesarean is 67% (8). In this regard, the American healthy people have targeted the rate of cesarean between 2010 and 2020, 15% in primiparous women and 63% in women with prior cesarean and VBAC rate up to 37% (3-4). 

In October 2016, the Iran’s Ministry of Health and Medical Education announced the VBAC clinical guidelines in accordance with the latest international guidelines (6). Nevertheless, the rate of VBAC in the first half of 2018 was less than 2% (1.73%) in Mashhad University of Medical Sciences (2). It seems that it is very low compared with countries with high rate of VBAC such as Ireland, Germany and Italy (29-36%) and also the Netherlands, Sweden and Finland (45-55%) and even in countries with low rate of VBAC such as US (10.1%) and Australia (19%) (7). Agency for Healthcare Research and Quality (AHRQ) (2006) suggested that instead of solely relying on biomedical criteria to determine whether a health intervention is necessary or successful, research should examine how individuals act and what information they provide about their experience of health care (8). 

It has been reported that not being informed properly and completely about the next birth options, confusing recommendations about birth options, the limited selection of supportive childbirth environments and the lack of emotional and psychological support provided to women have been known as the factors influencing women's decision about mode of birth after cesarean section (9). A recent study has suggested that 60% of women who had repeat cesarean section may not be aware of birth options (10). In addition, inadequate analgesia, professional organizations, and reluctance of childbirth providers have been reported as the related factors to low VBAC rate (11). Marrero (2012) believes that although maternal decision on cesarean in a large extent still depends on the patient's clinical condition, but studies have shown that individual manner of a physician’s performance significantly caused the differences in cesarean rates (4). There is an evidence of a difference in the rate of cesarean in public and private centers in societies (12, 13), including Iran (14). Therefore, as Munro (2016) suggests, it is necessary to investigate the barriers perceived by health care providers and decision makers to increase the access of women to VBAC (15). The purpose of this study was to explore the perceptions and experiences of clinicians and prior cesarean section mothers about the individual barriers to achieve VBAC.

## Methods

This study adopted qualitative content analysis with conventional approach as the method of inquiry. In this approach, the researcher tries to extract categories and themes from the data (16). Participants were selected using purposeful sampling with maximum variation. They included maternity health care providers, who had a rich experience of VBAC, consisting of gynecologists, midwives and health care providers at clinical, managerial and educational levels as well as mothers with previous cesarean section, who attended one of the women's hospitals in Mashhad in 2017 during pregnancy and postpartum (tables 1 & 2). In order to provide diverse experiences, participants had variety in terms of work experience, age, employment status, organizational position, occupational field (hospital/ health centers) and working experience in the private or public sections.

The informed consent form was signed by the participants after providing them the necessary information about the purpose of the study and ensuring the anonymity of the information and the confidentiality of the recorded interviews. Data collection was done by the first author (MF), who is an experienced midwife, through face to face semi-structured individual interviews and one focus group discussion. The mean duration of the individual interview session was 30-90 minutes and focus group discussion lasted 90 minutes. Data collection was conducted in hospitals and health care centers. An interview was initiated by a general question: Please talk about your understanding and experience of vaginal birth after cesarean section. Then more specific questions were asked: What barriers have you (as a gynecologist, midwife, and previous cesarean section mother) experienced regarding VBAC? Why maternal health care providers do not recommend VBAC to the mothers? Why mothers do not choose VBAC? What strategies could facilitate access to VBAC? Data collection and its simultaneous analysis was continued to 28 interviews when data saturation was obtained. Of those who were invited to the interview, only one physician declined the invitation due to work overload. Focus group discussion was arranged with 10 former interviewees (clinicians) after reaching data saturation in individual interviews with the aim of confirming the data collected already and giving feedback of the analyzed data to the interviewees. Each interview was transcribed verbatim as soon as possible and read through several times to get the insight of the whole story of the participants, then was analyzed by Graneheim and Lundman method (2004) (17) using MAXQDA10 software for data organization. In this way, the following steps were taken. Step 1: Identifying the unit of analysis. At this stage, the text of each interview was regarded as a unit of analysis. Step 2: Identifying the meaning unit as a part of the interview text, which contained one or more sentences or one paragraph with the same meaning. Step 3: Condensing the meaning units, abstracting them and labeling them as codes. Step 4: Grouping the total of primary codes in subcategories. Step 5: Continuing the process of induction and identification of the categories and finally the emergence of the theme. Credibility and authenticity was considered by the constant involvement of the researcher with the processes of data collection and analysis, frequently and repeatedly reading out the recorded interviews, planning a focus group discussion and receiving feedback about the accuracy of the data and conducted analysis. To maintain dependability and conformability of findings, help was received from external supervisors with qualitative research experience. Transferability of the study was achieved through adopting maximum variation strategy and purposeful sampling. Ethical considerations including the approval of the project by the Research Council affiliated to Mashhad University of Medical Sciences under code of 941372, as well as Research Ethics Committee with the code IR.MUMS.REC.1395.139, was taken into account. Obtaining informed consent, assuring the participants of the anonymity of the data and the freedom of participants to withdraw from the study at any time without prejudice was also considered. 

**Table 1:** The characteristics of Healthcare providers (n=21)

**Job status**	**N**	**Mean age**	**Mean of work experience(y)**	**Experience of VBAC**		**Number of years in ** **managerial position**
				**Yes**	**No**	
Obstetrician	6	45.33	15.33	6	-	2
Midwife (in health sector)	7	46.57	20.8	2	5	1
Midwife (in labor ward)	6	47.50	24.5	6	-	2
Other specialties	2	40	18	-	2	2

**Table 2:** The characteristics of mothers with previous cesarean section (n=7)

**Number of participant**	**Age**	**Education**	**Gravidity**	**Time since last delivery**	**Previous NVD**	**Occupation**	**Cause of previous CS**
6	38	Bachelor	2	2 years	No	Employed	Arrest of descent
7	35	Diploma	3	2 hours	Yes	Housewife	Multiple pregnancy
14	29	Master	2	pregnant	No	Employed	Decreased fetal movement
18	30	Associate Degree	2	3 years	No	Housewife	Arrest of dilation
19	36	Associate Degree	4	8 hours	yes	Housewife	Nonreactive NST
23	28	Associate Degree	1	5 years	No	Housewife	Mother’s request
26	34	Illiterate	2	pregnant	No	Housewife	Breech presentation

## Results

 The analysis of the clinician’s and women's perceptions and experiences towards the individual barriers of vaginal birth after cesarean (VBAC) revealed five sub-categories including: “sense of danger”, “financial displeasure”, “negative attitude”, “barriers to decision making” and “indolence”, which were set in two categories of “psychological barriers” and “ operational barriers”. At the end of analysis, “obstacles to acceptance and committed actions” emerged as the main theme of the study (table 3).

**Sense of danger: **The participants stated that fear of complications of vaginal birth after cesarean section, fear of legal accountability and fear of uterine rupture is a barrier to choose vaginal birth after cesarean. Mothers expressed their sense of danger in vaginal birth after cesarean section, including concerns about the health of the baby, fear of death during VBAC, and the non-assurance of birth safety.

*A mother with a history of VBAC said: *I went to the doctor and she said that you have to choose caesarean because you have had one cesarean section four years ago and it is possible that the sutures will break during the vaginal childbirth...!"

*An obstetrician and gynecologist with 25 years of experience, in relation to the complications of VBAC commented: *"The complication that the physician anyway knows about is uterine rupture. If rupture happens, it causes the mother to lose her uterus, lose the baby, and so on and you know, as a result physicians refuse to do it..."

**Financial displeasure: **This sub-category with the codes such as: lack of financial motivation for the physician in vaginal birth after cesarean section, lack of financial encouragement for midwife’s participation to the care of mother, and the lack of effectiveness of financial encouragement of the physician are the barriers in vaginal birth after cesarean section. 

*An obstetrician and gynecologist with 8 years of work experience in this relation said: *“The financial issue is not my hundred percent priority. The fee the doctor gets for vaginal birth after cesarean is higher than cesarean section, but this is not an incentive for the physician, because as compared to the time spent and the stress the physician endures, the wage is very low...”

**Negative attitude: **The negative attitudes toward vaginal birth after cesarean section was expressed in different aspects including: mother’s negative attitude towards skills of birth attendants, the birth environment, maternal safety in vaginal birth and VBAC outcomes. These are important barriers to vaginal birth after cesarean section. Mothers who have had cesarean section at their request and those who believed in vaginal birth after cesarean as a high-risk and challenging mode of birth, also did not choose VBAC.

*A midwife with 22 years of work experience in healthcare center showed her negative attitude in such a way: *“I don’t agree with vaginal childbirth currently. I believe that the complications of vaginal birth are high. I worked for a while in a private hospital, a famous doctor there told me: you have to manage a multiparous woman from dilation of 3 centimeters and a primiparous in the same stage, so that both of them get full dilated at 4 o'clock… when she came in the afternoon, both of them gave birth and then she came back. Because I worked in a private hospital, I believe the patient’s safety is not the target in many cases. Indeed, in the patient management process, late maternal complications are not important to them...”

*A midwife with 30 years of work experience in maternity hospital, similarly commented: *"...Whatever I saw here was not good at the end. That is, the patient has progressed even to the stage of full cervical dilatation and eventually has gone through cesarean section with a decrease in fetal heart rate or lack of or no progress of labor".

A mother with prior cesarean section declared her negative view as such: “…vaginal birth is not suitable for our country, these (physicians) cannot manage it properly! They tell us: I will come for your childbirth, but they don’t come. They don’t support mother in giving birth….”

**Barriers to decision making: **One of the most important barriers to achieve vaginal birth after cesarean section was to decide about the mode of birth by the mothers. Mothers had no competence in decision making due to the lack of awareness, and lack of effective consultation. They were affected by the cultural atmosphere including physician, spouse and those who influenced their choice and the misconceptions about mode of birth instead of choosing the mode of birth consciously.

*A pregnant mother with previous cesarean section said: *"...These days, vaginal birth has decreased to some extent, you know, women think that there is only one option for giving birth and that's cesarean section. Indeed the tendency of people to cesarean section has increased..."

*A midwife with 29 years of experience in healthcare center referring to the lack of right of decision making for the mothers commented: *"... Mothers don’t have the right to choose. Prior cesarean section mothers due to fear of vaginal birth and worries about the complications that may occur during birth, choose repeat cesarean. Most doctors do not accept VBAC and they tell to the women that you already had cesarean in your past pregnancy and now you have to choose repeat caesarean. In fact, mothers are forced to accept cesarean delivery because there is no support for the choice of vaginal birth after cesarean..."

*A midwife with 30 years of experience in maternity hospital concerning the role of most health professionals as barriers elaborated: *"Our mothers are not aware. If we talk a little bit with them, and say that your first birth was cesarean due to this and, you know, you are in good position now and you can do normal delivery and so on…. The next person come and say no, you cannot do it, you have had cesarean section, so vaginal birth is dangerous for you, it has complications, etc…and in such a way her opinion changes. Mothers are not so aware of making decision for themselves. If they analyze the situation and say that I had cesarean section in my first birth because of that reason but now I want to have vaginal birth…. Naturally, the midwife and the physician are the people who could change a mother’s mind…. Mothers don’t know that they could be responsible for decision making..."

**Indolence: **Participants explained the indolence of the maternity staff with terms such as convenience of cesarean section for a physician, lower stress with cesarean section, refusing to take care of the mother in VBAC, and less time and energy spend in caring of the mother. Also, birth management without any intervention in the process of VBAC is not compatible with the comfort of the staff during labor and birth. Besides Mothers, in their minds, provide more comfortable conditions for themselves and their relatives with scheduled cesarean. 

*An obstetrician and gynecologist with 29 years of work experience in this regard commented: *“…Even in our governmental centers, when the mother is going to do VBAC, physicians don’t accept her demand for vaginal birth. Here (university hospital), although senior supervisors worked before but now they do not spend enough time some of the midwives like the doctors seem lazy; they even do not spend time to talk to the patients ...”

*A midwife with 23 years of experience in managerial position indicated: *“Our doctors like to plan scheduled childbirth, it’s the most crucial problem. If we want the vaginal birth to begin spontaneously and end without intervention, certainly, it takes time and is not scheduled and it can occur at any time of the day and night and it may interfere with their plans, while cesarean section could be scheduled at a specified time by the physician ...”

*A 29 year old mother with prior cesarean section said:*

“In my opinion; some physicians disagree with VBAC for their own convenience…” 

**Table 3:** The codes, sub-categories, categories and theme emerged from data analysis

**Theme**	**Obstacle** **s to acceptance and committed actions **				
**Category**	**Psychological barriers**			** Operational barriers **	
**Sub-category**	**Sense of danger**	**financial displeasure**	**Negative attitude**	**Barriers to decision making**	**Indolence**
**Code**	Fear of VBAC safety	Lack of financial motivation for the physician	Unpleasant caring environment	Lack of effective consultation	Looking for convenience of CS by the physician
	Fear of legal accountability	Lack of financial encouragement for midwife’s	Not important for patient safety	Lack of awareness	Refusing to take care of the mother
	Fear of uterine rupture	The cost is overshadowed by other issues(mother)	Poor VBAC outcome	Being affected by the cultural issues	Less time and energy expenditure
	Concern about health of the neonate	Imbalance between the energy spent and the money earned	Negative perspective towards normal childbirth	unconscious selection of the delivery mode	More comfortable for mother and her relatives
	Fear of death during VBAC		Challenging perspective on VBAC		Ease in scheduling CS
	Non-assurance of birth safety				

## Discussion

Total rate of cesarean section and repeat cesarean are high in Iran and trial of labor after cesarean (TOLAC) is one of the important strategies to decline it. Despite the fact that it has been two years since the Ministry of Health of Iran has been issued the VBAC instruction, but clinical observations indicate that it is not being widely implemented. As a result of few cases of TOLAC, there are few studies about this topic in Iran. This article has explored the perceptions and experiences of health care providers and prior cesarean section mothers about individual barriers to VBAC. Based on the results, individual barriers towards VBAC included “sense of danger”, “financial displeasure”, “negative attitude”, “barriers to decision making” and “indolence”, which were set in two categories of “psychological barriers” and “operational barriers” and finally abstracted in the theme of “obstacles to acceptance and committed actions”.

“Sense of danger” was one of the individual barriers experienced both by the clinicians and mothers. Clinicians were quite afraid of complications of VBAC for the mother and her fetus and also had fear about their legal responsibility. Mothers with prior cesarean section was not also sure about the safety of VBAC and their uncertainty prevents them to make decision for VBAC. Marrero (2012) also reported the concerns about medical malpractice as one of the reasons for the lack of VBAC from the perspective of health care providers in her study (4). The sense of danger was also expressed by professionals and midwives in the study by Cox (2011); although they used several strategies for this barrier, but the most strategy was refusal of VBAC (18). NIH Consensus Development Conference Statement on VBAC (2010) reflects that the current legal medicine atmosphere including the understanding and experience of health care providers from professional litigation has an effect on preventing access to TOLAC (19). Chaillet et al. (2007) also stated that one of the barriers to implementing of clinical guidelines in VBAC was the fear of litigation in cases of uterine rupture (20). Keegan (2014), citing Declercq et al (2006), states: "One of the VBAC reduction factors is the defensive medicine in the sense of fear of medical malfunction, which acts as a driving force to suggest a specific mode of birth" (21).

Another individual barrier was the financial displeasure, because VBAC had no financial gain for midwives. In relation to the physicians, despite the fact that fee of VBAC is more than cesarean section, but physician’s higher responsibility and investment of more time for VBAC decrease the physicians’ motivation. Marrero (2012) also states that, time spent in caring of VBAC, and the imbalance between time spent and fee are reasons for physician’s unwillingness (4). 

In Canada, a specialist, a family physician and a midwife all receive the same fee, regardless of mode of birth. In the financial performance, there is no monetary motivation to perform cesarean section, similar to health management organizations of California that physicians are salaried or receive a steady amount regardless of birth (15). In fact, in our country, the financial incentive is inefficient because health care providers compare it to the time spent and factors such as psychological stress and acceptance of responsibility. Also for mothers, the choice of birth mode may be overshadowed by other issues besides its cost.

Negative attitude was another individual barrier for both mothers and clinicians. Mothers were reluctant to choose VBAC because of unpleasant experiences, inappropriate interaction of hospital staff during childbirth, unfavorable delivery environment and false beliefs regarding the safety of cesarean section. Maternal health care providers also considered VBAC as a challenging birth. They considered VBAC as a high risk birth with adverse outcome and having a negative attitude towards normal delivery, had a negative attitude toward VBAC. Chaillet (2007) reported the attitudes and beliefs of health care providers as well as mother’s motivation as barriers in implementation of clinical guidelines to decline cesarean section (20). Expectations and past experiences of mothers (22), previous birth experience, and personal meaning of birth (23), all affect the attitude of women toward birth.

In addition, decision making in the atmosphere of lack of informed choice for mode of birth was understood as another individual barrier to VBAC. Participants understood incompatibility of current cultural patterns with vaginal birth as a barrier to VBAC. While Renee (2012) reported that the sense of female control on the decision making process is one of the important factors to choose VBAC (3). In the study by Biraboneye (2017), two thirds of prior cesarean mothers had been informed of repeat cesarean section as the only method of birth. Although they referred to prenatal care centers more than three times, more than two thirds of them decided on repeat cesarean section. Another finding was a significant decrease in the choice of VBAC, after consultation regarding mode of birth. The researchers justified these findings because women’s decision making approach was significantly related to the preference of the consultant physician (24). Munro (2017) refers to the perception of health care providers about "women’s support in the choice of mode of birth" as an effective factor in access of women to VBAC. She believed that full information should be given about this choice and to be considered as experiences and emotions of mothers in their first childbirth, likewise hospital facilities for VBAC (15). Although specialists in Chaillet’s study (2007) stated that informing mothers about risks and benefits of TOLAC versus planned CS is time-consuming and may have little effect on the final decision, especially when women demand repeat cesarean section (20). Since the informed choice of mode of birth is in accordance with the rights of mothers, thus offering comprehensive counseling about this choice is an undeniable obligation for delivery team. Indolence was another barrier which was understood through the experiences of both health care providers and prior cesarean mothers as the individual barrier to VBAC. Doctors are exposed to less psychological stress in cesarean section and spend less time and energy by scheduling childbirth. Midwives also spend less time and energy to the care of mother in cesarean section. Because, the non-interventional approach in caring of mother in VBAC requires more time and energy. Bryant (2007) compared vaginal birth as an unpredictable and unorganized choice with cesarean section as a procedure with more confidence and discipline (25). In the study of Cox (2011) and also Eden (2004), both midwives and specialists described the comfort of scheduling repeat cesarean section (17, 26). Mothers have also more comfortable planning and better adjustment to their birth requirements by choosing repeat cesarean section. Aden (2004) found that not losing important events and the need of not being expected to begin labor, by choosing cesarean section, are the rationales of mothers in their desire to cesarean section (26).

In conclusion policy makers, healthcare planners, hospital administrators and maternity healthcare providers should plan to remove the individual barriers and identify facilitators to choose VBAC for mothers with prior cesarean delivery. Additionally, maternity healthcare providers should be encouraged to support this choice. Improving maternity care, shared decision making, create supportive environment (27) and the organization of VBAC care team (28) can help to promote VBAC rate and as a consequence decrease the rate of cesarean section. 
